# A new perspective on microRNA-guided gene regulation specificity, and its potential generalization to transcription factors and RNA-binding proteins

**DOI:** 10.1093/nar/gkae694

**Published:** 2024-08-16

**Authors:** Hervé Seitz

**Affiliations:** Institut de Génétique Humaine (UMR 9002), CNRS, 141, rue de la Cardonille, 34396 Montpellier, France

## Abstract

Our conception of gene regulation specificity has undergone profound changes over the last 20 years. Previously, regulators were considered to control few genes, recognized with exquisite specificity by a ‘lock and key’ mechanism. However, recently genome-wide exploration of regulator binding site occupancy (whether on DNA or RNA targets) revealed extensive lists of molecular targets for every studied regulator. Such poor biochemical specificity suggested that each regulator controls many genes, collectively contributing to biological phenotypes. Here, I propose a third model, whereby regulators’ biological specificity is only partially due to ‘lock and key’ biochemistry. Rather, regulators affect many genes at the microscopic scale, but biological consequences for most interactions are attenuated at the mesoscopic scale: only a few regulatory events propagate from microscopic to macroscopic scale; others are made inconsequential by homeostatic mechanisms. This model is well supported by the microRNA literature, and data suggest that it extends to other regulators. It reconciles contradicting observations from biochemistry and comparative genomics on one hand and *in vivo* genetics on the other hand, but this conceptual unification is obscured by common misconceptions and counter-intuitive modes of graphical display. Profound understanding of gene regulation requires conceptual clarification, and better suited statistical analyses and graphical representation.

## Graphical Abstract

**Graphical Abstract:**
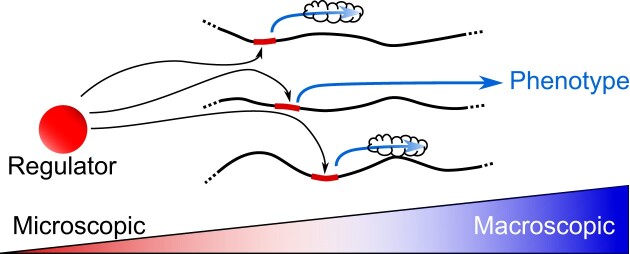

## Introduction: a shift in the definition of regulatory targets

The development of deep-sequencing-based methods allowed high-throughput identification of physical interactors for transcription factors (TFs) (1,2), microRNAs (miRNAs) (3) and RNA-binding proteins (RBPs) (4,5). Each of these analyses revealed that these gene regulators have very poor intrinsic specificity, with each TF, miRNA or RBP interacting with hundreds to thousands of distinct targets in mammals.

That discovery profoundly modified our representation of gene regulation. Before the advent of high-throughput methods, gene regulation was commonly described by a very restrictive ‘lock and key’ mechanism, where various biochemical discriminants would guarantee that the regulator physically binds to a very limited number of targets [see (6) for the example of transcriptional regulation]. In stark contrast with that view, gene regulation is now essentially presented as a complex, highly branched network, with each TF, miRNA or RBP controlling simultaneously a large number of genes whose coordinated (typically mild) regulation ultimately results in macroscopic phenotypes (7–10).

Yet, the biological functionality of many of these regulator–target interactions appears questionable: (i) A large fraction of experimentally identified binding sites for TFs (11,12), miRNAs (13) or RBPs (14) do not appear to be under selective pressure and are therefore not phylogenetically conserved. (ii) When a regulator controls a biological phenotype, and when the targets responsible for that phenotype are identified, these generally constitute a small minority of experimentally detected or phylogenetically conserved binding sites (see Table 1; such *in vivo* assessment of the functional contribution of individual binding sites has only been performed for miRNAs so far).

**Table 1.** Identified regulator–target interactions involved in biological phenotypes

Regulator	Functional target(s)	Reference(s)
*Mus musculus* miR-155	*AID* and *SOCS1*	(16,17,20)
*Cænorhabditis elegans* *let-7*	*lin-41*	(21,22)
*C. elegans* miR-791	*akap-1* and *cah-3*	(23)
*M. musculus* miR-200	*Zeb1*	(24)
*C. elegans* miR-35	*egl-1*	(15)
*Drosophila melanogaster* miR-iab-4/8	*hth*	(25)
*M. musculus* miR-140-5p	*Wnt11*	(18)
*Oryzias latipes* miR-202	*Tead3b*	(19)

In these experiments, the phenotypes of miRNA mutants were compared to the phenotypes caused by precise mutation of miRNA binding sites on individual targets. These studies found that abolishing the repression of one or two target(s) essentially phenocopies the miRNA mutant, suggesting that all the other targets contribute little to the organism-scale phenotype. The tested targets were usually selected by an educated guess, with the exception of *egl-1*, which was identified by an unsupervised screen among every predicted binding site for *mir-35* family miRNAs in *C. elegans* (15).

For four analysed miRNAs in Table 1, mutating these binding sites appeared to phenocopy every reported defect of the miRNA mutant. For the remaining four miRNAs, mutants in miRNA binding sites were reported to recapitulate most, but not all phenotypes observed in the miRNA mutant [class-switch recombination phenotype in *miR-155*^−/−^ B cells is not recapitulated in AID site mutant cells (16); Treg cell deficiency in *miR-155*^−/−^ mice is not recapitulated in SOCS1 site mutant mice (17); reduction in brood size and embryonic viability in *egl-1* site mutant *C. elegans* only partially phenocopies *mir-35* family mutants (15); some skeletal defects in *miR-140*^−/−^ mice are not recapitulated in *Wnt11* site mutant mice (18); decrease in medaka egg developmental success rate, as observed in *miR-202*^−/−^ females, is not recapitulated in *Tead3b* site mutants (19)]. These observations keep open the possibility that additional targets may also contribute to the observed phenotypes; alternatively, differences in genetic background between miRNA and target mutants, and miRNA-independent alteration of 3′ untranslated region (UTR) regulation, may explain differences between these mutants.

These observations deepen the paradox highlighted by (26) in the pre-deep-sequencing era, which was coined the ‘futility theorem’ (namely that almost every genomic occurrence of a sequence motif known to bind a TF is in fact devoid of a biological function). At the time, it was already clear that sequence motifs for any TF are highly frequent in metazoan genomes, while only ≈1/1000 of these sites were believed to be functionally important. It was proposed that chromatin structure and other factors could restrict site accessibility *in vivo*, therefore partially explaining that discrepancy (even though the theorem was referring to TFs only, that concept could easily be generalized to miRNAs and RBPs, whose RNA target sites can also be made inaccessible by RNA structures and by competing protein or RNA partners). Phylogenetic conservation was presented as an efficient filter in order to exclude unimportant sites.

Nowadays, after 20 more years of work (including high-throughput exploration of regulator binding *in vivo*), these notions can be revisited. Not only can the futility theorem be extended to other classes of regulators (at least miRNAs and RBPs), whose binding sites are also ≈6 bp long (hence similarly frequent in genomes), but it even applies to (i) binding sites that do bind the regulator efficiently *in vivo* and (ii) phylogenetically conserved binding sites.

Of note, the primary determinant for miRNA binding appears to be a perfect sequence match to the miRNA ‘seed’ (nucleotides 2–7 of the miRNA), potentially supplemented by additional context features (27). As a first approximation, it is therefore straightforward to predict whether a gene is a credible miRNA target, simply by verifying whether its 3′ UTR contains, or not, a perfect seed match. Such a ‘digital’ description of miRNA targets contrasts with the fuzzier prediction of TF or RBP targets, whose binding sites are more aptly described by a position weight matrix, which predicts molecular targets in an analogical manner.

The simplicity and clarity of miRNA target recognition greatly facilitated the functional exploration of miRNA–target interactions, which is more advanced than for TFs and RBPs. As a consequence, most of the data currently available on this topic deal with miRNA-mediated regulation, and most of the literature cited in this review centres on miRNAs. Another consequence is that the limitations in functional assignment for gene regulators are also better characterized for miRNAs [e.g. see (28,29)]. Yet, the concepts discussed here are formally the same for every class of regulator, and it can be anticipated that the TF and RBP literature will soon mature in the same direction as the miRNA literature does.

## Significance is not relevance

It therefore appears that most physical interactors for these gene regulators are not functionally affected by the regulator. This does not mean that their expression is insensitive to the regulator: many of these genes are actually regulated at the molecular scale, and transcription tends to be measurably affected by TF binding; RNA stability and translatability tend to be measurably affected by miRNA or RBP binding [e.g. see (30–34)].

However, such consequences at the microscopic scale do not necessarily translate into macroscopic phenotypes. Alterations in abundances of molecules do not constitute biological phenotypes: only phenotypes visible to natural selection (e.g. those affecting organismal fitness or reproductive ability of the kin) should be considered biologically relevant. That definition excludes traits that may be clinically relevant (e.g. phenotypes affecting lifespan after the end of reproductive age) or macroscopically visible (e.g. morphological variation that is neutral in selective terms), but whose effects would be inexistent on the species’ fitness.

Perhaps some confusion originated from sloppy usage of the term ‘significant’. Significance of a molecular effect [e.g. a change in messenger RNA (mRNA) abundance] means that the effect appears to be real, and (provided that the control conditions are adequate) it is unlikely to be due to confounding variables or random fluctuations in the experimental measurement. It certainly does not mean that the observed molecular effect has a biologically important function.

Changes at the microscopic scale do not necessarily trigger macroscopic effects visible to natural selection. First, the propagation of perturbations from the microscopic to the macroscopic scale is frequently buffered by homeostatic mechanisms, as exemplified by the large proportion of reported haplo-sufficient genes in animals (35–37), and, more generally, by the general robustness of biological phenotypes after small (≈2-fold) changes in gene expression (38,39). Second, even perturbations reaching macroscopic outcomes may be neutral in terms of natural selection (40).

Measured yet functionless changes in gene expression levels are often called ‘noise’. However, it is important to realize that functionless fluctuations in gene expression involve the same molecular actors (in particular, gene regulators) as biologically useful processes shaped by natural selection. It therefore appears meaningless to distinguish useless regulatory events from biological noise (8)—they do constitute ‘biological noise’. Similarly, it is misleading to name them ‘inadvertent regulatory events’ (8), as if there were ‘inadvertent’ versus ‘intentional’ biological processes: this is a finalistic description, and molecules do not have any intention—hence, from an objective point of view, functional and non-functional regulatory events are equally real and mechanistically indistinguishable, and they do not differ by any sort of intelligent desire.

## Advocating for intuitive data presentation

Graphical representation of the effect of a regulator on its molecular targets can influence the reader’s perception of the biological phenomenon, probably contributing to a misrepresentation of gene regulation efficiency. Here, I will discuss how the mode of graphical representation and the type of statistical tests being performed may alter the perception of the biological importance of gene regulation.

For example, endogenous miRNA-guided repression is very limited [typically, a few percent decrease in mRNA abundance, and only exceptionally >50% (13,30,31,41)]. However, for some reason, gene expression fold changes are frequently represented with poorly intuitive cumulative plots, rather than with the more familiar bar plots or box plots. Cumulative plots display (for any value of *x*) the proportion of genes whose fold change (or, more frequently, its logarithm) is lower than *x*. They typically exhibit a sigmoid shape, with very few genes having log(fold changes) lower than low *x* values, most genes having log(fold changes) lower than large *x* values and a steep increase around 0 because most genes have a log(fold change) close to 0.

**Figure 1.**
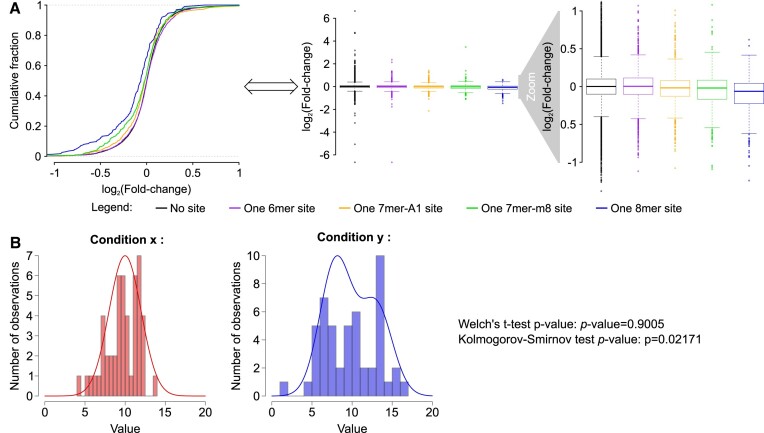
Graphical display and methodological choices can alter the reader’s perception of gene expression fold changes. (**A**) The same dataset (mRNA fold change 24 h after miR-9 transfection in HeLa cells) is shown as a cumulative distribution function plot (left) and as a box plot (right). (**B**) Two computationally generated datasets, centred on the same mean but differing by their shape (‘Condition *x*’ was sampled from a monomodal distribution and ‘Condition *y*’ from a bimodal distribution), were compared with a *t*-test (assessing the likelihood of equality of means in the sampled distributions) and with a Kolmogorov–Smirnov test (assessing any type of difference between the two sampled distributions).

Differences in fold changes that are so small that they are hardly visible on a box plot usually appear much more clear on a cumulative plot. This is exemplified by Figure 1A, showing the effect of miR-9 transfection on the abundance of its predicted target mRNAs in HeLa cells [dataset taken from (42)]. Candidate miR-9 target genes are stratified by miRNA complementarity site type [with 6-mer sites being the least efficient and 8-mer sites being the most efficient at guiding target repression (27)]. Cumulative curves are neatly distinct from each other, suggesting a clear difference in mRNA repression among targets with various site types. In contrast, a box plot of the same dataset yields highly overlapping boxes, clearly showing that the measured fold change values do not differ much among various site types. It is certainly preferable to use the more intuitive representation of gene expression fold changes with box plots, to make sure that the readership gets a correct intuition of the amplitude of gene regulation and how much it differs between various gene sets.

Unlike miRNAs (which all share the same effectors: Ago proteins, and their downstream effectors), the amplitude of regulation by TFs and RBPs can be quite diverse, reflecting the multiplicity of their mechanism of action. However, reported fold changes in the expression of their direct targets are also usually close to 1.5 (34,43–46). The mere fact that regulation strength can be variable among TFs and RBPs also implies that the extent of gene regulation by these factors should be made explicit: knowing that a gene is regulated is hardly useful if one cannot tell by how much it is regulated.

Besides graphical display, analytical strategies can also obscure the reader’s perception of gene regulation. In the scientific literature, it is now increasingly common to measure the significance of differences by performing a two-sample Kolmogorov–Smirnov test, rather than a *t*-test or its derivatives (Welch’s *t*-test, Wilcoxon test, Dunnett’s test or analysis of variance). Such a choice is hard to rationalize, because a statistical test should be chosen based on the specific hypothesis that is put to the test, irrespective of its apparent popularity in the field.

The Kolmogorov–Smirnov test assesses the likelihood that the two datasets are sampled from the same underlying distribution: a low *P*-value may be due to any sort of difference between the two distributions (mean, standard deviation or shape). On the other hand, a low *t*-test (or one of its derivatives) *P*-value allows us to conclude on the equality of the means of the two sampled populations, which is what the reader generally has in mind when assessing whether a regulator tends to have either a positive or negative effect on gene expression (see Figure 1B for a comparison: values for both conditions *x* and *y* tend to be close to 10, and a *t*-test on these datasets does not detect any significant difference, but distribution shape differs between *x* and *y*, and a Kolmogorov–Smirnov test finds a significant difference between those distributions). The Kolmogorov–Smirnov test is certainly useful in specific circumstances (e.g. when theoretical reasons suggest a difference in shape for the two distributions), but it is hard to interpret when assessing the effect of a regulator on its targets.

## Implications for functional assignment: with great sensitivity comes great responsibility

Because current high-throughput methods are so sensitive, it is now possible to detect trace amounts of nucleic acids and minuscule changes in molecule abundances. The fact that a molecule is observed does not imply that it is abundant enough to be functional (47). Before the development of such sensitive techniques, it was often implicitly assumed that an RNA abundant enough to be detected by Northern blotting, or a protein abundant enough to be detected by western blotting, was functional in that biological sample—hence the classification into ‘expressed’ and ‘non-expressed’ genes (in concrete terms, ‘expressed genes’ were those whose RNA or protein product yields a dark, neat band on a blot).

Now that deep-sequencing methods are available, it is likely that every RNA will appear to be expressed in any cell type, provided that its transcriptome has been sequenced deeply enough. Categorizing genes into ‘expressed’ and ‘non-expressed’ thus became meaningless, and such a qualitative notion is being replaced by a quantitative assessment (how much is this gene expressed in that sample?). Obviously then, functionality of a gene in a given biological sample can no longer be inferred from the fact that its expression has been detected—and there is no universal expression level threshold, above which a gene would play a biological function and below which it would not.

These notions are not specific to molecule abundance; they also generalize to biological processes (the fact that a protein is measurably phosphorylated does not imply functionality of that phosphorylation event, the fact that alternative splicing isoform distribution is modified does not mean that it plays a biological function, etc.).

In particular, physical interaction between molecules is frequently presented as a proof of functionality for that molecular complex. It should be stressed, however, how arbitrary the definition of a molecular complex can be: molecules keep interacting, more or less stably, and the detection of their interaction depends on experimental choices (salinity of buffers, temperature, etc.). Defining the composition of a complex simply means that the reported interactions were stable enough for co-purified molecules to be detected above some arbitrary cut-off, after being purified with arbitrarily stringent experimental conditions. With extreme stringency, complexes would be defined as single molecules, because all their interactants would have been undetected; with extreme sensitivity, the whole organism would appear as a giant complex (all the molecules eventually interact indirectly with each other, giving physical consistency to the whole body). Within that range, there is no natural stability threshold defining the biological functionality of complexes (e.g. codon–anticodon interactions are extremely weak, yet essential to every living organism). Consequently, the detection of a physical interaction between a regulator and a candidate target does not constitute a proof of biological functionality for that interaction.

## In vivo veritas

In order to confidently probe whether a regulator–target interaction plays a biological role, it is thus necessary to assess it *in vivo* (with ‘*in vivo*’ meaning ‘in the whole organism’, in contrast to ‘*ex vivo*’, meaning ‘in cultured cells’). Genome-editing techniques now allow precise mutation of regulators or their binding sites in individual targets, in a complete *in vivo* setting [e.g. for TFs, see (48); for miRNAs, see examples listed in Table 1].

When a binding site for a regulator has been mutated, there is always a risk that reported phenotypes are due to additional, unwanted effects of the mutation (e.g. mutating the site also perturbed a neighbouring binding site for another regulator, local chromatin organization, etc.). At least for miRNAs (whose target recognition rules are based on nucleic acid pairing, hence precisely predictable), a convincing control can help ruling out that possibility: mutating both the miRNA and the target site in a compensatory manner should restore that individual interaction and rescue those phenotypes that are due to that particular interaction (15,21,22).

When such *in vivo* data are not available, it is common practice to try to infer a regulator’s biological function from the automatically annotated functions of its molecular targets [e.g. Gene Ontology (GO) terms or Kyoto Encyclopedia of Genes and Genomes (KEGG) terms]. Comparing annotation terms for molecular targets and for the whole gene complement identifies terms that are over- or underrepresented in molecular targets, respectively to other genes. Significantly enriched or depleted terms among the numerous target genes for a TF or an miRNA are widely assumed to reflect that regulator’s biological activity.

That method suffers from major weaknesses: first, inadequate controls frequently generate false positives by systematic biases in the automatic annotation of predicted targets (49).

Second, many GO or KEGG terms do not distinguish agonists from antagonists (e.g. ‘regulation of endocytosis’ applies equally well to activators and repressors of endocytosis): the effects of a regulator on agonists and antagonists would partly cancel each other, and eventually the phenotypic effect could be weaker than that if a single gene in the pathway were regulated.

**Figure 2.**
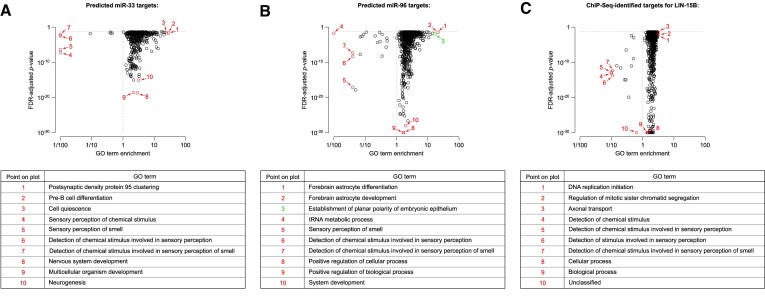
GO term enrichment is a poor predictor of a regulator’s biological activity. TargetScan-predicted targets for the miR-33 (**A**) and miR-96 (**B**) miRNA families, and ChIP-seq-identified targets for the LIN-15B TF (**C**) appear highly significantly enriched or depleted for several GO terms using the PANTHER overrepresentation test (in red and green: top-scoring GO terms, in terms of either fold change or *P*-value), yet most of them are unrelated to the observed *in vivo* phenotypes (in green: the only GO term which is related to the reported phenotype: point #3 in panel **B**).

Third, even though such enrichment analysis is widely used, it typically fails at predicting a regulator’s biological activity. Figure 2 shows a few examples where the actual genetic invalidation of a regulator allowed faithful assessment of its macroscopic effects. Knockout of the murine miR-33 miRNA triggers various changes in cholesterol and lipid metabolism (50,51), obesity and liver steatosis (52), and fibrosis (53). Mutation of the murine and human miR-96 miRNA triggers hearing loss (54,55), probably due to deficient differentiation of ciliated cells in the sensory epithelium (56). Mutation of the nematode *lin-15* locus, encoding the Lin-15A and Lin-15B TFs, perturbs vulval development (57). In these examples, enriched or depleted GO terms for candidate targets can rarely be linked to the reported phenotype: whether one selects the most enriched annotation terms (flagged with numbers 1–3), the most depleted terms (flagged with numbers 4–7) or the lowest enrichment *P*-values (flagged with numbers 8–10), the selected terms are usually unrelated to the actual phenotype (these terms are represented by red circles) and only exceptionally can they be logically linked (represented by a green circle).

Methodological improvements are therefore urgently needed. Biological functionality is safely assessed *in vivo* (e.g. after mutating regulator binding sites), while it cannot be extrapolated from molecular biology data and from poorly controlled computational tools and databases. The current scientific literature relies heavily on TF, RBP and miRNA databases, whose quality is sometimes questionable [e.g. see (58)]; on target prediction algorithms with high false-positive rates or strong dependence on experimental protocol details (59,60); and on pathway analysis methods that perform very poorly at predicting phenotypic outcomes (61).

It has been proposed that phylogenetically conserved binding sites reveal functional interactions whose biological advantage may be invisible in laboratory conditions (e.g. they only matter in rare environmental conditions) (62). In line with this view, comparative genomics has been heavily used in an attempt to identify biologically important interactions (63,64). Under the reasonable assumption that biologically important sequences are better conserved in evolution, much effort has been devoted in the identification of phylogenetically conserved binding sites for miRNAs, TFs or RBPs.

However, that approach is limited by the possibility that a sequence motif is conserved for other reasons, while fortuitously matching a consensus sequence for a regulator’s binding site (59). Because binding sites for miRNAs, TFs and RBPs all tend to be similarly sized (≈6 bp long in general), because there are only 4^6^ = 4096 distinct possible hexamers and because a few thousand regulators are known in the best annotated vertebrate genomes, chances are high for a given regulator binding site motif to be also recognized by other regulators—not even counting other sources of phylogenetic conservation (binding sites for the spliceosome on RNA, for chromatin fibre organization on DNA, etc.). It therefore appears that phylogenetic conservation of a candidate binding site is far from demonstrating biological functionality of the regulation, and it is certainly preferable that the burden of proof lies with whoever wants to assign a biological function to a given regulator–target interaction.

One major drawback of *in vivo* assessment is its low throughput. However, a large-scale screen for the biological importance of miRNA binding sites has already been performed for an miRNA family in *C. elegans* (15), and that method should be applicable to every genetically tractable species, as long as the phenotype of interest is clearly defined and easy to score.

Improvements in the identification of functionally important targets could also rely on a better understanding of the determinants of phenotypic sensitivity to gene expression levels. There is currently no high-throughput experimental method in metazoans for such a screen. However, proxies could be easier to measure: for example, assuming that dose-sensitive genes are tightly regulated (otherwise, their fluctuating expression could trigger adverse phenotypes), measuring interindividual variability in gene expression could identify genes with large fluctuations among wild-type individuals (these genes would be unlikely to be functionally sensitive to a moderate regulation) and genes with little interindividual variability (these genes would be the most promising candidates for phenotypically responding to a moderate regulation) (19,59).

This idea may also generalize to the cellular level: assuming that dose-sensitive genes are also tightly regulated among cells of a given cell type, single-cell RNA sequencing may reveal genes whose expression is most stable across cells, hence likely to be functionally sensitive to their expression level. Such an experiment faces several limitations (regarding the validity of its assumptions and regarding the relevant definition of a ‘cell type’, which may be artificial and arbitrary), but it is now readily doable for many species and tissues—time will tell how useful such results could be in the identification of functional regulatory targets.

## Towards a new definition of regulatory targets

It is possible to reconcile microscopic and macroscopic observations: if indeed regulators bind many targets at the molecular scale, while most of these interactions are inconsequential at the organism scale, it means that the consequences of most microscopic interactions are attenuated at the mesoscopic (i.e. intermediary) scale. And indeed, homeostatic mechanisms, which can efficiently buffer fluctuations due to external causes, must similarly attenuate changes of internal origin, like gene regulation.

Such mechanisms can involve negative feedback loops (e.g. if the pure effect of a regulator on a target gene is a 2-fold repression, a negative feedback could re-increase target expression and the final fold change may just be 1.5; hence, the regulation signal would be attenuated along its propagation from the microscopic to the macroscopic scale). They could also involve simple properties based on stoichiometry (e.g. in conditions where an enzyme is in large excess compared to its substrate, a small change in the enzyme’s expression would not have much of an effect on the biochemical flux because the enzyme would still be in large excess—meaning that the enzyme’s gene would be functionally insensitive to a small change in its expression level).

**Figure 3.**
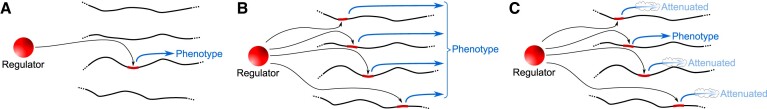
Models for regulators’ specificity of action. (**A**) In the historical, ‘lock and key’ model, gene regulators exhibit a great biochemical specificity. The few targets they regulate are responsible for the regulator’s biological phenotype. (**B**) In the current model, regulators coordinately control many targets, which collectively contribute to the phenotype. (**C**) In the newly proposed model, regulators exhibit poor biochemical specificity and many genes respond at the microscopic scale. However, homeostatic mechanisms attenuate the consequences of most of these events, and just a few regulator–target interactions actually contribute to the phenotype.

In contrast to the ‘lock and key’ model (where regulators interact with a limited set of targets; see Figure 3A), regulators would interact with large numbers of molecular targets. However, in contrast to the current, ‘coordinated network’ model (see Figure 3B), just a small number of interactions would have consequences propagating from the molecular to the organism scale, the other ones being attenuated by homeostatic mechanisms (see Figure 3C).

There is a natural antagonism between the mechanisms of developmental transitions (which tend to modify biological responses, e.g. cell differentiation or migration) and homeostasis (which tends to keep them constant). Both are defining features of life, but they have not yet been analysed under the same perspective. Quantitatively confronting these two phenomena therefore holds promises for the identification of biologically relevant regulator–target interactions: whenever a regulatory interaction is proposed, it is certainly useful to ask how efficiently its consequences can propagate from the microscopic to the organism scale, through various layers of homeostatic attenuation at the mesoscopic scale. From this point of view, it would be preferable to define ‘regulatory targets’ as the genes whose regulation triggers organismal consequences, rather than defining them by molecular properties (‘genes whose expression is affected’, ‘genes whose mRNA is physically bound by the regulator’, etc.).

## Conclusions

Technological advances in high-throughput molecular biology have revolutionized our perception of gene regulation, with evidence that most TFs, RBPs and miRNAs affect the expression of hundreds of genes each. That observation inspired the concept that regulators control biological phenotypes through the coordinated action of a large number of regulator–target interactions. The success of this theory may have been facilitated by disputable choices regarding graphical display and statistical testing of the effect of gene regulation, which fail to convey the information that gene regulation is usually modest in amplitude. At least when phenotypes are assessed at the organism scale, most of these interactions appear inconsequential, with just a few target genes seemingly responsible for regulator-controlled phenotypes.

Proper genetic invalidation of regulator binding sites, followed by an assessment of its macroscopic consequences *in vivo*, therefore appears indispensable for a convincing functional assignment of regulator–target interactions. It is important to realize that even *in vivo* data can be misleading, if the interaction was not abolished genetically. Genetic invalidation of a regulator’s binding site may not translate into any obvious macroscopic phenotype in laboratory conditions, even when abundant *in vivo* data suggested biological functionality [compare (65,66)].

In the argumentation in favour of the third model that I propose here (with homeostasis acting as a filter among consequential and inconsequential interactions), several limitations have to be addressed. It is likely that some interactions play biological roles in the wild (explaining why they have been conserved), while no phenotype has been reported in laboratory conditions when they have been experimentally mutated (62). It is also possible that some regulators control biological phenotypes through the coordinated regulation of many genes, yet such cases have not yet been observed because of the technical difficulty of experimentally manipulating many genes simultaneously (67).

At least this third model has the merit of reconciling molecular data and comparative genomics on the one hand and *in vivo* genetics on the other hand. It emphasizes the role of homeostasis (a universal feature in living organisms) in the convolution of regulatory signals. It also suggests that functionally important targets could be predicted more faithfully if the effects of regulatory network architectures were better understood, with perhaps specific network branches undergoing regulation attenuation through negative feedback loops, while other branches may undergo regulation amplification through positive feedback loops, ultimately leading to macroscopic phenotypes.

## Supplementary Material

gkae694_Supplemental_Files

## Data Availability

Scripts and intermediary data files used to generate Figures 1 and 2 are available at https://github.com/HKeyHKey/Seitz_2024 and https://www.igh.cnrs.fr/en/research/departments/genetics-development/systemic-impact-of-small-regulatory-rnas#programmes-informatiques.
